# A Machine Learning Method for Identifying Critical Interactions Between Gene Pairs in Alzheimer's Disease Prediction

**DOI:** 10.3389/fneur.2019.01162

**Published:** 2019-10-31

**Authors:** Hao Chen, Yong He, Jiadong Ji, Yufeng Shi

**Affiliations:** ^1^School of Statistics, Shandong University of Finance and Economics, Jinan, China; ^2^Institute for Financial Studies and School of Mathematics, Shandong University, Jinan, China

**Keywords:** Alzheimer's disease, differential networks, machine learning, neurodegenerative disease, gene expression

## Abstract

**Background:** Alzheimer's disease (AD) is the most common type of dementia. Scientists have discovered that the causes of AD may include a combination of genetic, lifestyle, and environmental factors, but the exact cause has not yet been elucidated. Effective strategies to prevent and treat AD therefore remain elusive. The identified genetic causes of AD mainly focus on individual genes, but growing evidence has shown that complex diseases are usually affected by the interaction of genes in a network. Few studies have focused on the interactions and correlations between genes and how they are gradually destroyed or disappear during AD progression. A differential network analysis has been recognized as an essential tool for identifying the underlying pathogenic mechanisms and significant genes for prediction analysis. We therefore aim to conduct a differential network analysis to reveal potential networks involved in the neuropathogenesis of AD and identify genes for AD prediction.

**Methods:** In this paper, we selected 365 samples from the Religious Orders Study and the Rush Memory and Aging Project, including 193 clinically and neuropathologically confirmed AD subjects and 172 no cognitive impairment (NCI) controls. Then, we selected 158 genes belonging to the AD pathway (hsa05010) of the Kyoto Encyclopedia of Genes and Genomes. We employed a machine learning method, namely, joint density-based non-parametric differential interaction network analysis and classification (JDINAC), in the analysis of gene expression data (RNA-seq data). We searched for the differential networks in the RNA-seq data with a pathological diagnosis of AD. Finally, an optimal prediction model was built through cross-validation, which showed good discrimination and calibration for AD prediction.

**Results:** We used JDINAC to derive a gene co-expression network and to explore the relationship between the interaction of gene pairs and AD, and the top 10 differential gene pairs were identified. We then compared the prediction performance between JDINAC and individual genes based on prediction methods. JDINAC provides better accuracy of classification than the latest methods, such as random forest and penalized logistic regression.

**Conclusions:** The interaction between gene pairs is related to AD and can provide more insight than the individual genes in AD prediction.

## 1. Introduction

With the improvement of the standard of living, people's life expectancy has gradually increased, but at the same time, the aging population is also growing, and age-related diseases such as dementia are on the rise (1). Dementia is a condition that causes a severe loss of cognitive abilities due to a disease or injury. Dementia caused by a traumatic brain injury is usually static, whereas dementia caused by neurodegenerative diseases is usually progressive and may eventually become fatal (2). Alzheimer's disease (AD) is the most common cause of dementia and the most common neurodegenerative disorder (3). The clinical features of AD are a decline in memory or other thinking skills that affect a person's ability to perform daily activities. AD is a complex and chronic neurological disease, affecting more than 200,000 people younger than 65 years old and 5 million people older than 65 years old. At present, the total estimated prevalence is expected to be 13.8 million (4). AD is a critical public health issue in many countries around the world, with a significant health, social, and financial burden on society (5).

At the neuropathological level, AD is characterized by progressive cortical atrophy due to neuronal loss and characteristic intracellular and extracellular deposits of insoluble tau and amyloid-β (Aβ) proteins (6). Although the pathology and molecular mechanisms of AD have been explored through various methods such as gene expression profiling, genome-wide association studies (GWAS), or a systems biology framework, the cause of AD is still unclear (7–11). AD is a multifactorial disease, including age, genetic factors, excess use of alcohol, or depression (5). Among these factors, genetic factors can explain an estimated 70% risk of AD (12). Thus far, a lot of GWAS have shown that many risk loci and genes are related to AD. A dominant mutation in the gene encoding presenilin-1 (PSEN1), presenilin-2, amyloid precursor protein, and apolipoprotein E (APOE) is an identified genetic cause of AD (13). However, current works have mainly focused on the genetic variations of individual genes associated with AD. Few studies have focused on the interactions and correlations between gene products and how they are gradually destroyed or disappeared during AD progression.

The interactions of genes can be adequately represented as a network (14, 15). Accordingly, a differential network analysis can be used to identify different structures between the gene networks of two specific groups. Generally, these specific groups of networks are often represented as the patient group and healthy control group. Through the differential network analysis, we can identify whether the connectivity of a particular set of genes of interest, or a given single gene has changed between two networks (16–23). The differential network analysis can be used to understand the effects of different genes and to identify interactions between essential genes that affect AD. In the past few years, the differential network analysis has been one of the techniques that has drawn researchers' attention and has become an active field of research. For example, the method DEDN, proposed by Zhao et al. (24), uses a precision matrix to build two models of each condition-specific network under the Gaussian assumption. Yuan et al. (25) also proposed NES in 2016, which is the first method to detect group differences between pathways. This method can find changes in edges and nodes and consider the pathway structure. However, depending on the characteristics of AD, not all differential network analysis methods are suitable. Many confounding factors are involved in AD, such as age, gender, and depression, which are all related to the progression and development of the disease. Korolev (5) also confirmed that confounding factors can affect AD. To identify the differential patterns of gene product network activation more accurately between the patient group and healthy controls, we should select the method that can cope well with these confounding factors. Moreover, in analyzing a specific disease, the use of network biomarkers to achieve accurate classification is meaningful, especially in high-dimensional settings. Furthermore, in AD, the probability distribution of gene product measurements may be unknown, so we should select a method that does not have the parametric probability distribution hypothesis of gene product measurements. To the best of our knowledge, thus far, no one has proposed a method to solve all of the above issues at the same time.

To address the challenges above, we propose the use of a novel machine learning method, namely, joint density-based non-parametric differential interaction network analysis and classification (JDINAC), which was proposed by Ji et al. (26). The JDINAC method eliminates the effects of confounding factors in the differential network analysis and uses high-dimensional sparse data for an accurate classification. Moreover, JDINAC makes no assumptions about the probability distribution of the gene measurement parameters. In this study, we first employ JDINAC in the analysis of high-dimensional “omics” and autopsy data from the Religious Order Study (ROS) and Memory and Aging Project (MAP), two well-known studies in the area of AD research. Then, we selected genes from the AD pathway of the Kyoto Encyclopedia of Genes and Genomes (KEGG) and searched for potential biomarkers and differential networks of gene expression data with a pathological diagnosis of AD. Finally, we obtained an optimal prediction model that was subsequently built through a cross-validation. Such a model showed good discrimination and calibration for AD prediction.

## 2. Materials and Methods

### 2.1. ROS and MAP

Data were obtained from two famous cohort studies in the area of AD: the ROS and MAP. Both were proposed by Bennett (27, 28). ROS is a longitudinal clinical pathology cohort study of AD. Since 1994, the study has recruited people over the age of 65 from more than 40 groups (nurses, pastors, and siblings) in the United States. As a complementary study of ROS, MAP is also a longitudinal clinical pathology cohort study that focuses on cognitive and motor function decline and AD risks. The project began in 1997 and enrolled older people (65 years old and above) from retirement communities in Chicagoland and Northeastern Illinois. These studies are both run by Rush University and approved by Rush University Medical Center Institutional Review Boards. In addition, participants in both studies did not have known dementia at the time of enrollment and agreed to receive annual clinical assessments and to donate their brain after death. For the present study, we used a subset of the ROS and MAP datasets that includes a reduced selection of more commonly used variables (i.e., clinical diagnosis, demographics, and RNA-seq).

### 2.2. Clinical Diagnosis

Based on a three-stage process, a clinical diagnosis of AD status was proposed, including scores of computerized cognitive tests, clinical judgment of neuropsychologists, and diagnostic classification of clinicians.

All participants were required to have a unified, structured clinical assessment, including a set of 19 cognitive tests. These tests were scored by a computer using a decision tree, which was designed to simulate clinical judgment. Then, neuropsychologists, who were blinded to the demographics of the participants, examined injury ratings and other clinical information and made clinical judgments on the presence of injuries and dementia. Lastly, a clinician (neurologist, geriatrician, or geriatric nurse practitioner) reviewed all available data, examined the participants, and presented a final diagnostic classification. The final diagnostic is divided into six groups: NCI (no cognitive impairment), MCI (mild cognitive impairment and no other causes of cognitive impairment), MCI+ (mild cognitive impairment and another cause of cognitive impairment), AD (AD and no other causes of cognitive impairment), AD+ (AD and another cause of cognitive impairment), and other dementias (other primary causes of dementia).

In this study, we examined the effects of gene pairs on AD. To ensure the accuracy of our data analysis, we selected samples without cognitive impairment or with AD but without other causes of cognitive impairment. Accordingly, we eliminated the unclear effects of other cognitive impairments on our research. The final samples were selected from two diagnostic groups, namely, NCI and AD.

### 2.3. Demographics

Basic demographic information includes age, gender, and years of education, and in this study, they represent confounder covariates. Age is calculated based on the dates of birth and death. Gender and years of education were self-reported from the baseline evaluation.

### 2.4. RNA-Seq

RNA was extracted from the dorsolateral prefrontal cortex gray matter of the ROS and MAP datasets using QIAGEN's miRNeasy mini kit (catalog number 217004) and the RNase-free DNase kit (catalog number 79254). They were quantified by NanoDrop and evaluated for quality by Agilent Bioanalyzer. The Broad Institute's Genomics Platform uses the strand-specific dUTP approach (29) with poly-A selection (30) on samples to prepare the RNA-seq library. All samples were selected to meet two initial quality criteria: a quantity threshold of 5 g and a RNA integrity (RIN) score >5. Raw RNA-seq data were processed by parallel automated pipelines [see (31) for the details of the RNA-seq data pipeline].

### 2.5. Study Design

In this study, to ensure that the variables were included in all of the samples, we selected 365 samples from the ROS and MAP databases, including 193 clinically and neuropathologically confirmed AD subjects and 172 NCI controls. The RNA-seq data need to be obtained from the gray matter of the dorsolateral prefrontal cortex. We therefore emphasize that, although the ROS and MAP are cohort studies, the 365 samples were deceased. We randomly selected 135 subjects from the AD group and 120 subjects from the NCI group as our training samples. A total of 255 training samples and 110 test samples were therefore used.

We extracted 171 genes from the AD pathway (hsa05010) of KEGG and treated these genes as our candidate genes. We then filtered the genes that contained a >30 % zero gene expression value in our data. A total of 158 final candidate genes were therefore used for the analysis.

### 2.6. Differential Gene Co-expression Methods

A differential network analysis is a standard method used to discover differences in a network topology between two groups of gene expression samples. In this study, we employed the differential network analysis to detect the differential interaction patterns of the genes selected between two specific groups (i.e., AD and NCI) and to build a classification model using these genes. However, the differential network analysis and the classification in our research are confronted with some challenges. First, the number of features *p* is often much bigger than the sample size of data *n*, and here, *p* is the number of pairs of genes. Second, nonlinear relationships often appear in the analysis of two genes. Third, AD may be affected by confounding factors, such as age, gender, and years of schooling. Therefore, we have to address these confounding factors in a differential network analysis and classification. Lastly, due to the difficulty in obtaining the underlying distribution of genes, some specific distribution assumptions often fail, such as the Gaussian assumption. To address the above challenges, we compared various differential network analysis approaches. We then selected the most suitable method for our study, which is JDINAC (26). We employed this newly proposed machine learning model, that is based on a non-parametric kernel approach, to recognize differential interaction patterns of genes and to find gene pairs that are most closely related to AD. We then built a classification model using these gene pairs. In the following text, we briefly introduce the JDINAC method.

The main premise of JDINAC is that the difference in the gene network between patients with AD and healthy people arises from the collective effect of differential pairwise gene–gene interactions. Here, through a nonparametric kernel method, we estimate the conditional joint density of pairs of genes in different groups and characterize them as the pairwise gene–gene interactions. Formally, we denote ${\text{X}}_{n\times p}={\left({\text{X}}_{1},{\text{X}}_{2},\ldots ,{\text{X}}_{n}\right)}^{\text{T}}$ as the matrix of *n* samples and *p* genes and ${\text{Y}}_{n\times 1}={\left({\text{Y}}_{1},{\text{Y}}_{2},\ldots ,{\text{Y}}_{n}\right)}^{\text{T}}$ as the response vector. We denote *s* (*s* = 1, 2, …, *n*) as the individual participant. Then, X_*s*_, *s* = 1, 2, …, *n*, represents the gene features in the *s*-th people. We define Y_*s*_ as the binary response variable, which can be represented as:

(1) $$\begin{array}{l} {\text{Y}}_{s}=\left\{ \begin{array}{cc} 0, & \text{if }s\text{ is non-AD}\\ 1, & \text{if }s\text{ is AD} \end{array}\right. \end{array}$$

Pr denotes the probability of the patients with AD, i.e., Pr = ℙ(Y_*s*_ = 1), and G_*i*_ is the *i*-th gene. Based on the logistic regression, JDINAC can be built as

(2) $$\begin{array}{l} \text{logit}\left(\text{Pr}\right)=\alpha_{0}+\overset{T}{\underset{t=1}{\sum }}\alpha_{t}{\text{Z}}_{t}+\overset{p}{\underset{i=1}{\sum }}\underset{j>i}{\sum }\beta_{ij}\text{ln}\frac{f_{ij}^{1}\left(G_{i},G_{j}\right)}{f_{ij}^{0}\left(G_{i},G_{j}\right)},\\ \overset{p}{\underset{i=1}{\sum }}\underset{j>i}{\sum }|\beta_{ij}|\leq c,c>0 \end{array}$$

where *Z*_*t*_(*t* = 1, 2, …, *T*) indicates the covariates, such as age, gender, and depression, which is used to adjust the confounding factors. We define $f_{ij}^{1}\left(G_{i},G_{j}\right)$ as the class conditional joint density of the *i*-th gene (*G*_*i*_) and *j*-th gene (*G*_*j*_) in Class 1, i.e., $\left(\left(G_{i},G_{j}\right)|Y=1\right)\sim f_{ij}^{1}\left(G_{i},G_{j}\right)$ and $f_{ij}^{1}\left(G_{i},G_{j}\right)$ represents the strength of the association between *G*_*i*_ and *G*_*j*_ in Class 1. Similarly, we define $f_{ij}^{0}\left(G_{i},G_{j}\right)$ as the class conditional joint density of the *i*-th gene (*G*_*i*_) and *j*-th gene (*G*_*j*_) in Class 0, i.e., $\left(\left(G_{i},G_{j}\right)|Y=0\right)\sim f_{ij}^{0}\left(G_{i},G_{j}\right)$ and the $f_{ij}^{0}\left(G_{i},G_{j}\right)$ represents the strength of association between *G*_*i*_ and *G*_*j*_ in Class 0. The parameters β_*ij*_ denote the differential dependency patterns between condition-specific groups (32).

As this is a high-dimensional problem, we need to adopt the *L*_1_ penalty (33). Therefore, to estimate β_*ij*_, we solve *L*_1_ penalized logistic regression, and to obtain the best penalty parameter, we employ a cross-validation method. The estimation procedure of JDINAC includes a multiple splitting procedure and a prediction averaging procedure, which guarantee robust and accurate results. For the multiple splitting procedure, the data are split into two parts. The first part data are used to estimate the kernel density functions ${\hat{f}}_{ij}^{1}\left(G_{i},G_{j}\right)$ and ${\hat{f}}_{ij}^{0}\left(G_{i},G_{j}\right)$, whereas the second part data fits the *L*_1_ penalized logistic regression. For the prediction averaging procedure, we repeat the first procedure for pre-determined times [for the detailed implementation of JDINAC, please refer to (26)].

## 3. Results

### 3.1. Characteristics of the Subjects in This Study

Among the 365 subjects in this study, 193 were diagnosed with AD and 172 subjects had NCI. The demographics of the two groups of subjects (i.e., AD and NCI) are presented in Table 1. In the table, no statistically significant difference was found between the two groups with respect to female sex ratio and years of education (p > 0.05). However, statistically significant differences in the APOE ε4 ratios and age at death between the two groups were detected (p < 0.001). A higher ratio of subjects who had APOE ε4 in the AD group than the NCI group is found.

**Table 1:** Sample demographics of the subjects included in this study.

	**AD**	**NCI**	
**Variable**	**(*N* = 193)**	**(*N* = 172)**	***p*-value**
Female sex, No. (%)	133 (68.9)	103 (59.9)	0.072
Education, years	16.54 (3.42)	16.48 (3.43)	0.876
APOE ε4, No. (%)	69 (35.6)	28 (16.3)	<0.001
Age at death, years	88.29 (3.08)	84.60 (5.32)	<0.001

*Data are presented as mean (SD) unless specified*.

### 3.2. Differential Gene Co-expression Patterns

**Figure 1:**
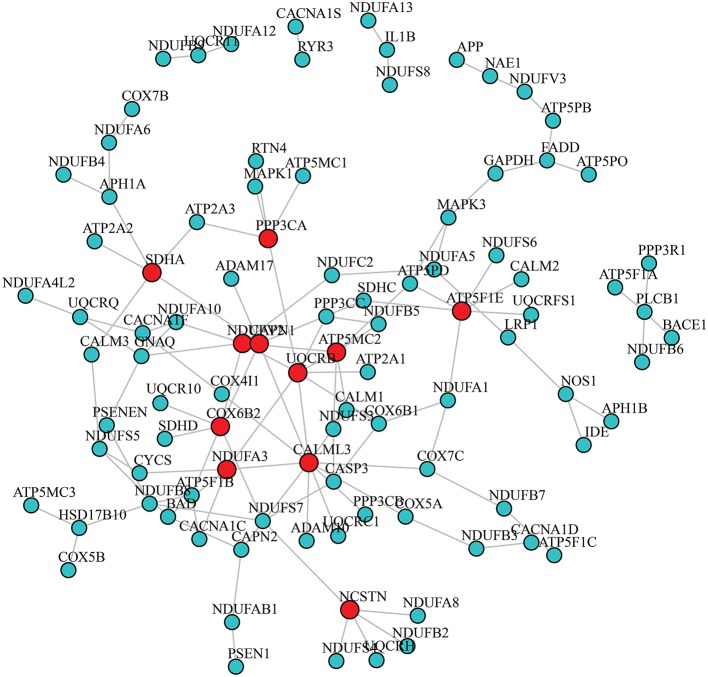
The differential network of AD pathway between AD subjects and NCI subjects. An edge presented in the differential network means the relation of corresponding pair genes is different between two condition-specific groups. The red nodes stand for hub genes.

After adjusting for the covariates, the differential gene co-expression network of AD estimated by JDINAC is described in Figure 1. In this figure, we did not show all 171 genes; instead, we chose genes connected with at least one other gene. A total of 114 edges are shown in the figure, which meant that 114 pairs of genes are associated with a pathological diagnosis of AD. In the figure, the red nodes represent the hub genes that have at least five adjacent genes in the differential networks. Hub genes included 11 genes: CALML3, UQCRB, NDUFV2, ATP5MC2, COX6B2, ATP5F1E, CAPN1, NCSTN, SDHA, NDUFA3, and PPP3CA. We ordered these hub genes based on the weight derived by JDINAC of genes. We mainly focused on the top 10 differential gene pairs identified by JDINAC, which are summarized in Table 2.

**Table 2:** Top 10 differential gene co-expression pairs identified by JDINAC.

	**Gene 1**	**Gene 2**
1	UQCRB	NDUFV2
2	NDUFV3	ATP5PB
3	NDUFB8	PSENEN
4	NDUFV2	NDUFA10
5	NDUFS8	IL1B
6	CAPN2	NDUFAB1
7	PSEN1	NDUFAB1
8	NDUFC2	NDUFA5
9	PPP3CC	NDUFB5
10	ATP5MC2	NDUFB5

### 3.3. Prediction Performance

**Figure 2:**
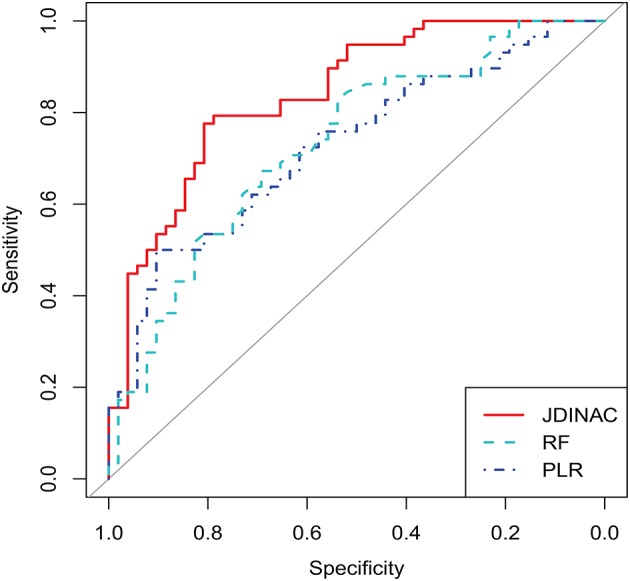
ROC curves for JDINAC, penalized logistic regression, and Random Forest.

In this section, we demonstrate that our model has good predictive performance by examining whether individual genes or pairwise interactions of genes are stronger associates of AD. Accordingly, we compared JDINAC and two popular methods in single-gene prediction: random forest (RF) and penalty logistic regression (PLR). We used all 158 individual genes as predictors in the RF and PLR methods. The evaluation of their prediction performance is shown in Table 3. We present three receiver operating characteristic curves in Figure 2.

**Table 3:** Evaluation and comparison of prediction performances of Random Forest and Penalized Logistic Regression based on individual genes and JDINAC based on pairwise interactions of genes.

	**JDINAC**	**RF**	**PLR**
AUC	0.840 (0.763–0.916)	0.731 (0.637–0.825)	0.727 (0.630–0.824)
Accuracy	0.791 (0.716–0.866)	0.682 (0.604–0.760)	0.673 (0.591–0.755)
Sensitivity	0.776 (0.636–0.916)	0.672 (0.417–0.928)	0.724 (0.459–0.989)
Specificity	0.808 (0.652–0.963)	0.692 (0.431–0.954)	0.615 (0.355–0.876)
Precision	0.818 (0.705–0.931)	0.709 (0.542–0.876)	0.677 (0.510–0.845)

*95% confidence interval are presented in parentheses*.

As shown in Table 3 and Figure 2, the two methods with individual genes (i.e., RF and PLR) performed worse than JDINAC in terms of the area under the curve (AUC) and accuracy. Moreover, compared with two other methods, JDINAC not only showed higher sensitivity and specificity but also had a better balance between them. Thus, the interactions between the pairs of genes are associated with AD and can provide more insights than the individual genes in AD prediction. These results suggest that identifying differential networks is biologically meaningful for distinguishing between disease states and normal disease states.

Furthermore, to prove that our method (i.e., JDINAC) is more stable than the other two methods (i.e., RF and PLR), we calculate the 95% confidence intervals for all the indicators through the bootstrap method and list them in Table 3. The results show that JDINAC has a smaller confidence interval than the other two methods, thus showing its stability. Furthermore, JDINAC is significantly better than the other two methods in terms of predictive power. We used the DeLong test (34) to compare the AUC value of JDINAC with the AUC values of RF and PLR. Statistically significant differences in the two AUC values between JDINAC and RF and between the JDINAC and PLR were also detected (*p* = 0.016 and *p* = 0.007, respectively). The findings show that the AUC index of JDINAC is significantly improved compared with that of the other two methods with a confidence level of 0.05.

## 4. Discussion

### 4.1. Key Findings

The goal of the current study is to determine the underlying genetic interaction mechanisms of AD and use these identified network genes to achieve accurate classification. At first, we believed that the relationship between gene expression and AD is complex and considering the response of AD to any individual genes is insufficient to fully capture or interpret this relationship. By selecting 158 genes from KEGG as our final candidate genes, we identified 114 pairs of genes that were related to AD through the use of JDINAC. The analysis of the relationship between AD and gene expressions suggested that the key influencing factor of AD is the interaction between genes. In other words, AD is rarely the result of a single genetic abnormality, but it rather reflects the various genes that interact in the network. Further analysis revealed a differential network in the pairwise interactions between a limited number of genes that predict AD more accurately and strike a good balance between sensitivity and specificity.

### 4.2. Interpretation

We identified the differential interaction patterns of a network of gene pairs between the AD and NCI groups. Then, we identified 11 hub genes and the top 10 important gene co-expression pairs. Although the real underlying dependence relationships of genes are still unknown, the association of these gene pairs and AD can be supported by the results of previous studies. Among the 11 hub genes, seven were mitochondrial genes, which are located in the mitochondria. Moreover, the 10 gene co-expression pairs included five gene pairs that are mitochondrial genes, and 15 genes among the 20 genes are located in the mitochondria. Mitochondrial genes are therefore essential for AD, and considerable literature has already confirmed this finding (35–37). Two specific examples for a gene pair and a hub gene that are supported by previous studies are discussed below.

The first example, UQCRB, is a panthenol-cytochrome c reductase binding protein. It is a nucleus-encoded component of complex III, which is located in the mitochondrial respiratory chain. This protein plays an essential role in the electron transfer as a complex of ubiquinone and QP-C. Scientists have shown that the composition of complex III is regulated in the early onset of AD (38). Therefore, UQCRB will affect AD progression. NDUFV2 is a protein encoding a subunit of the mitochondrial respiratory chain complex I. Similarly, complex I and complex II transfer some electrons to ubiquinone. Complex III uses ubiquinol to reduce cytochrome c (39). Therefore, UQCRB has a functional interaction with NDUFV2. This finding suggests that the molecular role of UQCRB in the AD progression is derived from the altered UQCRB-NDUFV2. Another example is the hub gene ATP5F1E, which is located in the mitochondria and encodes a subunit of the mitochondrial ATP synthase. Cha et al. (40) found that the ATP synthase subunit α (ATP5A) was O-GlcNAcy at Thr432, whereas ATP5A O-GlcNAcy-lation was reduced in the brain of patients with AD and transgenic mouse models. This finding means that ATP synthase is associated with AD. Therefore, ATP5F1E plays a crucial role in the progression of AD.

The association of pairs of non-mitochondrial genes with mitochondrial genes in the differential network and AD can be confirmed by previous research findings. For instance, PSEN1 and NDUFAB1 are identified as a pair affecting AD and PSEN1 are not located in the mitochondria. PSEN1 is a protein-coding gene and makes a protein called presenilin-1. NDUFAB1 is a protein encoding a subunit of the mitochondrial respiratory chain complex I. In PSEN1, mutants often directly or indirectly lead to an increase in mitochondrial calcium ion Ca^2+^ content. The increased Ca^2+^ content in the mitochondria stimulates mitochondrial respiration leading to an increase in mitochondrial superoxide production (41). PSEN1 therefore has functional interactions with NDUFAB1. As mitochondrial dysfunction and subsequent metabolic disorders are observed in AD and changes in the PSEN1–NDUFAB1 gene pair affecting the mitochondrial function, we can speculate that a correlation exists between the PSEN1–NDUFAB1 gene pair and AD. In addition, we provide another example for a hub gene that is not located in the mitochondria. As shown in Figure 1, the hub gene CALML3 has the largest number of neighbor genes. The above analysis shows that Ca^2+^ can affect AD. Strehler (42) already found that the CALML3 gene is involved in the transport of Ca^2+^. Therefore, the gene CALML3 will affect the onset of AD.

### 4.3. Future Prospects

The main aim of this study is to identify the underlying gene interaction mechanisms of AD. Although the study conducted thorough research, certain limitations were still encountered. These limitations are expected to be addressed in our future studies.

First, a lot of potentially essential genes do not belong to the AD pathway of KEGG, which may affect the accuracy of our study. In the future, we can use the GWAS dataset to investigate the association of gene interaction and AD. Second, our analysis only identified paired gene interactions, however, the relationship between AD and gene interactions may be more complex. In the future we should therefore modify the method that identifies higher-order interactions of genes. Furthermore, because of the limitation of the ROS and MAP datasets, we could not divide the subjects of the AD group into more specific stages of AD, including early mild cognitive impairment, late MCI, and AD. In the future, we can perform a differential co-expression analysis across all the stages of AD and derive detailed results.

### 4.4. Conclusion

In this study, we employed JDINAC for the estimation of gene co-expression networks of AD. Our findings showed a strong association between AD and gene interactions, and we can predict AD through the patterns of interactions within the gene network. By comparing JDINAC with RF and PLR, we found that the interactions between pairs of genes had more information than the individual genes in AD prediction. In the future, further optimizations of this study will be conducted to provide more accurate results and to discover a broader range of applications.

## Data Availability Statement

The datasets [ROSMAP] for this study can be found in the [Synapse platform] and the accession number is Synapse: syn3219045 [https://doi.org/10.7303/syn3219045].

## Ethics Statement

The studies involving human participants were reviewed and approved by Institutional Review Board of Rush University Medical Center. The patients/participants provided their written informed consent to participate in this study.

## Author Contributions

HC, YH, and JJ were responsible for conceptualization and methodology. JJ contributed to data analysis. HC and YH contributed to writing of the original draft. HC, YH, JJ, and YS contributed to the writing of the review and editing.

### Conflict of Interest

The authors declare that the research was conducted in the absence of any commercial or financial relationships that could be construed as a potential conflict of interest.
